# Odor-Free Lyophilized Trout (*Oncorhynchus mykiss*) Powder in Gluten-Free Pasta: Nutritional, Techno-Functional, Sensory and Digestibility Evaluation

**DOI:** 10.3390/foods15071155

**Published:** 2026-03-28

**Authors:** Özlem Emir Çoban, Hülya Gül, Mücahit Eroğlu, Tuba Okutan, İlhan Firat Kilinçer, Feray Çağiran Yilmaz

**Affiliations:** 1 Fisheries Faculty, Fırat Üniversity, Elazig 23119, Türkiye; 2 Department of Food Engineering, Faculty of Engineering and Natural Sciences, Suleyman Demirel University, Isparta 32000, Türkiye; 3 Faculty of Science, Firat University, Elazig 23119, Türkiye; 4 Digital Forensics Engineering, Firat University, Elazig 23119, Türkiye; 5 Department of Nutrition and Dietetics, Faculty of Health Sciences, Dicle University, Diyarbakir 21280, Türkiye

**Keywords:** gluten-free pasta, trout powder, deodorization with vinegar, lyophilization, buckwheat flour, sensory acceptance

## Abstract

Gluten-free (GF) products developed for individuals with celiac disease and gluten sensitivity often suffer from low protein and mineral content. Fish proteins offer a promising solution to address these deficiencies; however, the characteristic “fishy odor” and related technological challenges limit consumer acceptance. This study aimed to develop an innovative GF pasta with improved nutritional density and acceptable sensory quality by incorporating deodorized and lyophilized trout powder. GF pasta formulations were prepared using buckwheat flour, xanthan gum, and 5% or 10% odorless trout powder. Vinegar pretreatment was applied to reduce fish odor, while lyophilization was chosen to minimize nutrient losses. The samples were analyzed for nutritional composition, techno-functional properties, in vitro digestibility, and sensory attributes. Results showed that trout powder significantly increased protein and ash content compared to the control (*p* < 0.05). A slight darkening was observed in color analysis due to fish pigments and buckwheat phenolics, but overall visual stability remained high. In vitro digestibility revealed enhanced protein digestibility (*p* < 0.05) and a slight reduction in starch digestibility. Sensory evaluation demonstrated that odor scores (8) at 10% trout inclusion remained close to the control, reversing the commonly reported decline in acceptance with increasing fish content. These findings indicate that combining vinegar pretreatment with lyophilization enables the incorporation of fish proteins into GF pasta without sensory disadvantages, while simultaneously improving nutritional quality.

## 1. Introduction

Pasta is among the most widely consumed staple foods worldwide due to its low cost, ease of preparation, and long shelf life. Traditionally produced from durum wheat semolina, pasta provides energy through its high carbohydrate content but remains limited in essential amino acids such as lysine and threonine [1]. In recent years, the increasing prevalence of gluten-related disorders, including celiac disease and gluten sensitivity, has driven consumers toward gluten-free (GF) diets. However, GF pasta formulations based on starch-rich raw materials such as corn, rice, or sago often exhibit poor nutritional profiles, characterized by low protein, fiber, and mineral contents. This nutritional gap has intensified the search for innovative ingredients that can enhance both the technological quality and nutritional value of GF products [2].

Fish proteins represent a promising candidate for food enrichment due to their balanced amino acid composition, high digestibility, and the presence of omega-3 fatty acids (EPA and DHA), which are critical for cardiovascular health [3,4]. Rainbow trout (*Oncorhynchus mykiss*), in particular, offers high biological value and is well-suited for incorporation into pasta matrices. However, the biggest obstacle to including fresh fish-derived ingredients in food formulations is the characteristic “fishy” odor caused by volatile compounds. Previous studies have consistently reported that increasing levels of fish meal in pasta formulations significantly reduce sensory acceptability, often leading to consumer rejection [2,3].

Various methods exist in the literature to remove fishy odor; the use of antioxidants, washing, or masking with spices are some of them. In this study, however, vinegar (acetic acid) was employed as a deodorization agent, offering a distinctive approach to the chemical elimination of volatile compounds in fish muscle. Organic acids not only facilitate the dissociation of odor-binding molecules from proteins but also neutralize the basic amine compounds responsible for fishy odor, thereby stabilizing the aromatic profile of the product. Furthermore, lyophilization (freeze-drying) was applied to the trout powder, a technique that minimizes protein denaturation compared to thermal drying methods, ensuring superior preservation of nutrients and minerals [5].

A review of the current literature reveals that while studies exist on the use of different fish species in gluten-free pasta formulations, the effect of deodorized lyophilized trout powder and its contribution to the in vitro protein digestibility of this ingredient have not yet been investigated. The primary aim of this research is to evaluate, through an integrated approach, the nutritional composition, techno-functional properties, sensory profile, and protein digestibility of GF pasta enriched with vinegar-deodorized lyophilized trout powder. This study not only provides a high-value alternative for individuals with gluten sensitivity but also establishes a sustainable and innovative model for the incorporation of fish proteins into food matrices in a sensory-acceptable manner.

## 2. Materials and Methods

### 2.1. Materials

The rainbow trout (*Oncorhynchus mykiss*) used in this study were obtained from the processing and packaging facility of Altaş Su Ürünleri Ltd. Şti (Altınordu, Ordu, Türkiye). The fish were transported fresh in insulated polystyrene boxes containing ice and delivered under hygienic conditions to the Processing Technology Laboratory of the Faculty of Fisheries, Fırat University. Buckwheat flour, employed as the primary raw material in gluten-free pasta production, was purchased from a commercial supplier. All analytical procedures were carried out using reagents of analytical grade purity to ensure accuracy and reproducibility. In particular, pepsin (≥2000 U/mg protein; Sigma-Aldrich, St. Louis, MO, USA) was used in the gastric phase, while pancreatin (standardized to trypsin activity, 8.4 U/mg protein; Sigma-Aldrich) was applied in the intestinal phase. For the multi-enzyme pH-stat technique, trypsin (1.6 mg/mL) was employed. The source, activity units, and application conditions of each enzyme have been clearly described to guarantee methodological transparency and reproducibility.

### 2.2. Methods

#### 2.2.1. Deodorization of Fish Mince

The deodorization process of trout mince was adapted from Emir Çoban [6] with modifications. Fresh rainbow trout (*Oncorhynchus mykiss*), weighing approximately 2000–3000 g, were transported to the laboratory under aseptic conditions. The fish were cleaned by removing tails, viscera, skin, fins, and bony structures, followed by washing, draining, and mincing (5 mm plate diameter, Philips Viva Collection HR2726/90 Meat Grinder, Amsterdam, The Netherlands). To ensure consistency and representativeness of the raw material, the mince was prepared from the entire muscle mass of the fish rather than selected portions. This approach minimizes variability between samples and provides a standardized basis for subsequent deodorization and analytical procedures.

To eliminate odor components, the trout mince was immersed in a vinegar solution prepared at a concentration of 10 v/100 v (vinegar/ultrapure water). During this period, the mince was manually stirred for approximately 1–2 min every 10 min to ensure uniform contact with the vinegar solution. At the end of this treatment, the mince was filtered using a cheesecloth bag. The filtered mince was spread in sterilized trays at a thickness of 2 cm and frozen at −20 °C for 24 h. Freeze-drying was subsequently performed at −55 °C for 48 h under a vacuum pressure of 20 Pa. The deodorized freeze-dried trout mince was initially ground using a laboratory-scale knife mill (GRINDOMIX GM 200, Retsch, GmbH, Haan, Germany). The ground material was then sieved through a stainless-steel laboratory sieve (20–30 mesh, corresponding to approximately 250–600 µm), which is the particle size range typically used for durum wheat semolina in pasta production. This sieving step ensured uniform granule size. The standardized mince was stored in oxygen-impermeable, light-proof bags at 4 °C until further use.

#### 2.2.2. Preparation of Gluten-Free Functional Pasta with Odor-Free Lyophilized Fish Powder

Gluten-free pasta was prepared according to the AACC 66-41.01 procedure [7,8]. Buckwheat flour served as the primary raw material, supplemented with 1% xanthan gum and water. In the experimental production, deodorized lyophilized trout powder (RT) was incorporated at substitution levels of 5% (5% RT) and 10% (10% RT), replacing an equivalent proportion of buckwheat flour. Together with the control, three different pasta types were produced. The dough was mixed in the kneading chamber of a pasta machine for 12–15 min to ensure homogeneous hydration. Hydration was adjusted to reach a final moisture content of 30% (±2), as specified in the AACC 66-41.01 procedure. The extruded pasta was shaped into ribbon-like strips (about 1 mm thick, 3 mm wide, and 80 mm long), resembling shortened tagliatelle. This design was chosen to ensure uniform cooking and facilitate sensory evaluation. To prevent sticking and preserve shape, the pasta samples were placed on cloth-covered wire racks and rested at room temperature for about 10 min. Drying was then carried out in a hot-air oven at 50 °C with a relative humidity of ~65%, until the moisture content reached ~10% (dry matter basis). The dried pasta was then packaged under modified atmosphere conditions (60% CO_2_ and 40% N_2_).

#### 2.2.3. Proximate Composition Analyses

The nutritional composition of deodorized lyophilized trout powder and cooked pasta samples was determined according to AOAC [9] methods. Prior to analysis, the dried pasta samples were ground into fine powder using a laboratory mill and stored in airtight containers at room temperature until testing. Moisture content was analyzed using the oven-drying method (Method 950.46), fat content was quantified by Soxhlet extraction (Method 960.39), crude protein (dry weight basis) was measured by the micro-Kjeldahl method (Method 928.08), and ash content was determined by incineration (Method 920.153). Carbohydrate content was calculated by difference according to the standard proximate analysis method AOAC [9]. Specifically, the sum of moisture, protein, fat and ash was subtracted from 100%.

Energy values of the pasta samples were calculated by multiplying protein and carbohydrate contents by 4 kcal/g and fat content by 9 kcal/g, followed by summation of the results, and expressed as kcal/100 g [10].

#### 2.2.4. Techno-Functional Properties of Pasta

##### Optimum Cooking Time

Optimum cooking time was determined according to AACC [8]. Pasta samples were cooked in a beaker, and after 6 min, a piece was removed at 1 min intervals using forceps and pressed between glass plates. The cooking time was recorded as the point at which the central opaque core disappeared.

##### Weight, Volume Increase, and Swelling Index

Weight increase was determined by cooking 20 g of pasta in 250 mL of boiling distilled water for the optimum cooking time. After cooking, the pasta was drained and weighed. The difference between raw and cooked samples was expressed as a percentage according to the following formula [10]:

$$\mathrm{Weight}\;\mathrm{increase}\;(\%)=\;\frac{WC-Wr}{Wr}\;\times \;100$$

Volume increase was assessed following the method of Cleary and Brennan [11]. Three grams of pasta were cooked in 180 mL of distilled water for the estimated optimum cooking time, cooled in 100 mL of cold water, and the change in volume was recorded. Volume increase (%) was calculated using the following equation.

$$\mathrm{Volume}\;\mathrm{increase}\;(\%)=\frac{VC-Vr}{Vr}\times 100$$

where VC is the weight of the cooked pasta and Vr is the weight of the raw pasta. Subsequently, the cooked pasta was dried in an oven at 105 °C until constant weight (24 h). The swelling index (SI) was calculated as:

$$\mathrm{SI}\;(g/g)=\;\frac{Wc}{Wd}$$

where Wc is the weight of the cooked pasta (g) and Wd is the weight of the oven-dried pasta (g).

##### Cooking Losses

Cooking loss was determined by cooking 3 g of pasta in 180 mL of water for the optimum cooking time [12]. The cooking water was collected, evaporated at 105 °C until constant weight, and the residue was expressed as a percentage of the initial pasta weight.

##### Color Analysis

The color of the pasta samples was determined using a colorimeter (Model CR-400, Konica Minolta Sensing, Inc., Osaka, Japan). Lightness (L), redness (a*), and yellowness (b*) values were recorded, with each measurement repeated at least three times. In this system, L* represents brightness, a* indicates red when positive and green when negative, while b* reflects yellow when positive and blue when negative [12].

##### Texture Profile Analysis (TPA)

Texture profile analysis of pasta samples (5 g) was performed according to the procedures described by Hayıt et al. [13] and Acun and Gül [14]. Pasta samples were cooked to their optimum cooking time, and four ribbon-like pieces (approximately 3 mm wide and 80 mm long) were placed side by side on the base plate. Since the individual strips were smaller than the cylindrical probe (2 cm diameter), they were aligned together to fully cover the probe surface. Compression tests were then performed using this probe, which compressed the samples twice to generate compression–relaxation–stress profile curves. The analysis parameters were: pre-test speed 1 mm/s, test speed 5 mm/s, post-test speed 5 mm/s, and a load cell capacity of 5 kg.

#### 2.2.5. In Vitro Digestion and Glycemic Response

In vitro gastrointestinal digestion was conducted following the INFOGEST 2.0 protocol [15]. Homogenized powder samples (5 g) were mixed with 3.5 mL of simulated salivary fluid (SSF) and supplemented with 25 µL of 0.3 M CaCl_2_·2H_2_O. The ratio of food to SSF was adjusted to 1:1 (5 g food: 5 mL SSF) with ultrapure water, and the mixture was incubated at 37 °C, 100 rpm for 2 min.

Preparation of Simulated Fluids:

- SSF (salivary fluid): KCl 15.1 mM, KH_2_PO_4_ 3.7 mM, NaHCO_3_ 25 mM, NaCl 0.15 mM; adjusted to pH 7.0.
- SGF (gastric fluid): NaCl 6.9 mM, KCl 0.9 mM, KH_2_PO_4_ 0.5 mM; adjusted to pH 3.0.
- SIF (intestinal fluid): KH_2_PO_4_ 6.8 mM, NaCl 105 mM, NaHCO_3_ 30 mM; adjusted to pH 7.0.

All pH values were calibrated and verified after preparation.

For the gastric phase, 8 mL of SGF was added together with 5 µL of 0.3 M CaCl_2_·2H_2_O. The pH was adjusted to 3 using 3 M HCl, and 1 mL of pepsin solution (≥2000 U/mg protein; Sigma-Aldrich) was added. The mixture was diluted to 20 mL (food:SGF = 1:1) with ultrapure water and incubated at 37 °C, 200 rpm for 2 h.

For the intestinal phase, 11 mL of SIF was added to the gastric digest. The pH was adjusted to 7 with 1 M NaOH, followed by 40 µL of 0.3 M CaCl_2_·2H_2_O, 2.5 mL bile solution (final concentration: 10 mM bile salts), and 5 mL of pancreatin solution standardized to trypsin activity (8.4 U/mg protein; Sigma-Aldrich). The final volume was adjusted to 40 mL (food:SIF = 1:1) with ultrapure water. Samples were incubated at 37 °C, 150 rpm for 2 h.

At the end of intestinal digestion, samples were centrifuged at 4000 rpm for 10 min at 4 °C. Supernatants were filtered through 0.45 µm filters, collected in clean tubes, and digestion was terminated by adding 9 µL of 500 mM Pefabloc enzyme inhibitor to 1 mL of sample. The supernatants were subsequently analyzed for protein digestibility (%), and glycemic response (%).

Protein digestibility was calculated based on the release of free amino groups measured by the OPA method:

$$\mathrm{Protein}\;\mathrm{Digestibility}\;(\%)=\;\frac{Free\;amino\;groups\;after\;digestion\;(OPA)}{Initial\;total\;protein\;content}\;\times \;100$$

Glycemic response was determined by monitoring glucose release during digestion and calculating the area under the curve (AUC):

$$\mathrm{Glycemic}\;\mathrm{Response}\;(\%)=\;\frac{AUC\;(Sample)}{AUC\;(reference\;glucose)}\;\times \;100$$

Here, AUC (sample) represents the area under the glucose release curve of the digested sample, while AUC (reference glucose) corresponds to the area obtained from pure glucose under the same conditions.

#### 2.2.6. Sensory Evaluation

In this study, sensory analysis was carried out with 20 panelists who had prior experience in the sensory evaluation of fish products. Before the assessment, panelists attended a briefing session that covered sample size, consumption procedure, sensory contact (smelling, chewing), and preference/acceptance criteria. Pasta samples were cooked to their optimum cooking time (AACC; Method 66-50.01) [8] in boiling water containing 1% salt and 1% vegetable oil, and were served hot, individually, and without any accompaniments, in accordance with ISO 13299:2020 [12]. During the evaluation, whole-grain diet biscuits and water were provided to neutralize palate perception [12].

Sensory evaluation was performed using a nine-point hedonic scale (1 = dislike extremely, 9 = like extremely) for homogeneity, characteristic color, fish odor, sour aroma, firmness, elasticity, pasta-specific aroma, and fish aroma [16,17]. Since the scale is ordinal, results were expressed as median values.

This study was approved by the Non-Interventional Research Ethics Committee of Fırat University, and informed consent was obtained from all participants prior to sensory testing.

#### 2.2.7. Statistical Analysis

All experiments were conducted in triplicate, and the results were expressed as mean ± standard deviation. Statistical analyses were performed using SPSS 16 (Version 16, IBM Corp., Armonk, NY, USA). One-way analysis of variance (ANOVA) was applied to determine significant differences among formulations. When ANOVA indicated significance, Tukey’s multiple comparison test was used to identify differences between group means. For sensory data, differences among samples were analyzed using the Kruskal–Wallis test, and when significant differences were observed, the Dunn post hoc test was applied. A confidence level of 95% (*p* < 0.05) was considered statistically significant.

## 3. Results and Discussion

### 3.1. Proximate Composition

The addition of odorless lyophilized trout powder (RT) significantly increased the protein, ash, and fat contents of gluten-free pasta samples compared to the control group (*p* < 0.05) (Table 1). This increase can be attributed to the high protein and mineral composition of trout powder, which directly enriches the formulation. In line with previous studies, the incorporation of tuna flour (9%) raised the protein content of sago pasta from 1.40% to 4.90%, while the use of salmon powder (20%) increased protein levels in fresh pasta up to 23.40% [17,18]. Yerlikaya et al. [19] reported that lyophilization preserves mineral content more effectively than oven-drying, particularly for Na, P, Ca, and Mg, and this preservation advantage explains why trout powder provides superior protein and mineral enrichment compared to other fish powders. Although mineral composition analysis was not performed in the present study, the observed increase in ash supports the stabilizing effect of lyophilization on nutrients. Furthermore, the rise in fat content can be explained by the natural lipid components of trout powder. Consistently, Ainsa et al. [20] found that pasta enriched with sea bass by-products contained 2.89% fat in durum wheat and 4.80% in spelt formulations. These findings confirm that seafood-derived ingredients are effective in enhancing the nutritional value of gluten-free pasta and align with the results of our study.

In this work, the decrease observed in the total carbohydrate content of pasta enriched with odorless lyophilized trout powder is fully consistent with the “starch–protein substitution” phenomenon reported in the literature (Table 1). The use of animal protein sources such as rainbow trout (*Oncorhynchus mykiss*) in place of plant-based flours naturally reduces the overall starch proportion in the food matrix. The present findings confirmed that increasing levels of fish powder led to a reduction in carbohydrate content (*p* < 0.05). Aínsa et al. [20] reported that the carbohydrate content of pasta enriched with sea bass by-products decreased to 62.43% (durum) and 51.59% (spelt). Similarly, Ainsa vd. [21] and Monteiro et al. [22] found that fish protein concentrate reduced carbohydrate content. These results are in line with our observations.

The carbohydrate profile of gluten-free pasta ingredients is also critical. Raw materials such as sago starch or Mocaf, which are highly carbohydrate-rich (73–90%), can lead to high glycemic loads. The incorporation of fish powder into such gluten-free formulations has been highlighted as a key strategy to balance this nutritional profile [1,2,23,24].

The energy value of pasta is calculated from the sum of protein, lipid, and carbohydrate contributions.

In the current study, the energy value of the control sample was 264.4 kcal, while the 5% and 10% trout powder-enriched groups reached 272.42 and 280.25 kcal, respectively (*p* < 0.05). Desai et al. [25] reported that the addition of 20% salmon powder increased the energy value of cooked pasta from 122.26 kcal to 161.08 kcal/100 g due to higher lipid content. Monteiro et al. [22] also observed an increase from 167.97 to 187.89 kcal with tilapia flour enrichment. Conversely, Goes et al. [26] found that tilapia protein concentrate reduced the energy value from 252.71 to 236.58 kcal/100 g, which was explained by a sharp reduction in carbohydrate content (4 kcal/g) despite protein enrichment.

The energy value obtained in the present study indicate that trout powder enrichment increased nutrient density without excessively raising energy density. Commercial gluten-free pasta available in the European market is generally reported to range between 338–384 kcal/100 g [27]. For example, Garofalo gluten-free farfalle and linguine contain 356–361 kcal/100 g, while Barilla gluten-free tagliatelle provides 364 kcal/100 g (Nutracheck Database). These values confirm the reported range and demonstrate that pasta prepared with deodorized lyophilized trout powder offers a safer option for celiac patients, providing a balanced profile in terms of calorie control while simultaneously enhancing nutritional quality.

Color is one of the most critical quality attributes influencing consumer acceptance of pasta [28,29]. In this study, the incorporation of odorless lyophilized trout powder into gluten-free buckwheat pasta led to distinct changes in lightness (L). The 5% RT group showed a decrease in L values, resulting in a darker appearance, whereas the 10% RT group exhibited an increase in L*, producing a lighter tone. The darkening observed in the 5% RT group can be explained by the limited transfer of fish pigments into the phenolic-rich buckwheat matrix, which reduces light reflection. In contrast, the lightening effect in the 10% RT group may be attributed to the higher level of trout powder, which promotes a more homogeneous distribution of pigments, combined with the stabilizing effect of lyophilization. Ramya et al. [16] and Yerlikaya et al. [19] emphasized that freeze-drying preserves both mineral and pigment stability more effectively than oven-drying, supporting the interpretation that pigment stabilization at higher enrichment levels contributed to the lighter appearance.

Total color difference (ΔE) is a key index reflecting how enriched products are visually perceived compared to the control group. In the literature, ΔE values in fish-enriched pasta typically range between 3 and 12 [1,22,30]. In the present work, the ΔE values obtained (6.45 for 5% RT and 6.58 for 10% RT) fall within this range, confirming perceptible differences detectable by consumers. Yerlikaya et al. [19] highlighted that ΔE values above 5 are perceived as noticeable changes, while Monteiro et al. [22] suggested that such trends may strengthen consumer associations with “whole wheat pasta” or “naturally enriched functional products,” although excessive fish flour incorporation could lead to a grayish tone and reduce acceptability. Importantly, unlike tuna or tilapia powders that often impart grayish or brownish hues, trout pigments are stabilized during freeze-drying, producing controlled reddish-yellow tones. This stabilization not only enhances visual appeal but also distinguishes trout powder as a more favorable option for gluten-free pasta enrichment.

The results further indicate that reddish (a^*^) and yellowish (b^*^) tones decreased in enriched samples compared to the control. This reduction can be attributed to the interaction of trout pigments with the phenolic compounds of buckwheat flour and the specific color profile of salmonid species. While Desai et al. [18] reported that salmon powder imparted a reddish hue to pasta, and Pangestu et al. [3] observed a brownish-yellow color with tuna flour addition, our findings suggest that trout powder, despite pigment stabilization during freeze-drying, interacts differently with buckwheat, leading to a more muted color profile. Ramya et al. [16] and Yerlikaya et al. [19] confirmed that freeze-drying is superior to oven-drying in maintaining pigment stability, which explains why the observed changes remained controlled rather than producing undesirable grayish tones.

**Figure 1:**
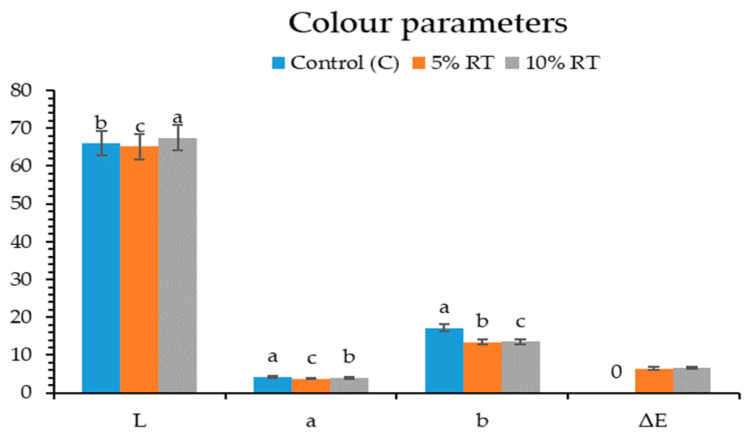
Color parameters (L, a, b) and overall color difference (ΔE) of gluten-free pasta enriched with odorless lyophilized rainbow trout powder. ^a, b, c^: There is a statistically significant difference between the values within the color parameters that are denoted by different letters (*p* < 0.05).

Overall, the color changes observed in buckwheat-based gluten-free pasta enriched with trout powder can be attributed to the combined effects of pigment transfer, matrix interactions, and the stabilizing role of freeze-drying (Figure 1). Importantly, the ΔE values obtained (>5) exceed the threshold for perceptible differences, confirming that the changes are visually detectable and may directly influence consumer acceptance. These findings align with previous studies showing that seafood-derived ingredients not only enhance the nutritional profile of gluten-free pasta but also significantly alter its visual attributes, which must be considered in product development and consumer evaluation.

The technological and in vitro digestibility properties of gluten-free pasta enriched with odorless lyophilized rainbow trout powder are presented in Table 2. The findings indicate that fish powder supplementation had significant effects on both sets of properties (*p* < 0.05).

Weight and volume increase were highest in the control sample, whereas the addition of RT led to a reduction. This outcome is consistent with previous reports suggesting that fish proteins, together with lipids, interfere with starch hydration during cooking, thereby limiting gel formation [18,19,30]. Regarding the swelling index, the 5% RT sample showed values comparable to the control, while a decrease was observed at 10% RT. This reduction supports earlier findings that protein enrichment restricts starch granule swelling [17,21].

Cooking loss in gluten-free pasta was approximately 4.06–4.16% in both the control and fish-supplemented samples. Although slight increases were observed in the fish-enriched groups compared to the control, these differences were not statistically significant (*p* > 0.05). This result indicates that the addition of lyophilized fish did not increase the loss of soluble components in gluten-free pasta, thereby preserving the cooking stability of the products. In contrast to previous reports [31,32], where higher cooking losses were observed in fish-enriched pasta, our study demonstrated lower values, highlighting the stabilizing effect of lyophilized fish addition.

Conversely, cooking time increased with higher RT levels. This suggests that protein addition delayed starch gelatinization and slowed water penetration. Comparable results were reported by Desai et al. [18], who observed prolonged cooking times in fish protein-fortified pasta.

From a nutritional perspective, the most remarkable finding was the reduction in glycemic response (%). While the control sample exhibited a glycemic response of 61.21, the value decreased to 47.28 with 10% RT supplementation. This reduction can be attributed to the ability of proteins and lipids to interfere with starch digestion, thereby lowering glycemic impact. In addition, the starch content of the pasta formulations decreased proportionally with the incorporation of trout powder, since fish-derived ingredients replaced part of the starchy raw material [25,31].

Finally, in vitro protein digestibility significantly improved with RT addition. The control sample showed a digestibility of 76.79%, which increased to 84.34% in the 10% RT sample. This enhancement is explained by the high bioavailability of fish protein. Laishram et al. [32] demonstrated that fish protein hydrolysates improve digestibility in pasta, while Ainsa et al. [21] reported similar improvements in pasta enriched with fish by-products. Moreover, Desai et al. [25] highlighted that fish proteins and lipids can interact with starch and protein matrices in pasta, enhancing nutrient bioavailability and reducing starch digestibility. Monteiro et al. [22] also confirmed that fish protein supplementation in pasta modifies technological properties and contributes to nutritional fortification. In contrast to previous reports [30,31], where higher cooking losses were observed in fish-enriched pasta, our study demonstrated lower values, highlighting the stabilizing effect of lyophilized fish addition. In addition to protein digestibility, trout powder incorporation also contributed to nutrient release during digestion, as indicated by the increased ash and fat contents in the formulations (Table 1). This suggests that minerals and lipids became more available, consistent with the findings discussed in Section 2.2.5.

### 3.2. Texture Profile Analysis (TPA)

The addition of odorless lyophilized rainbow trout powder to gluten-free pasta resulted in clear modifications in its textural profile (Table 3). Hardness and chewiness were highest in the control sample, while both parameters decreased with fish protein supplementation (*p* < 0.05). This reduction can be explained by the dilution of starch and the formation of a softer protein matrix. Govinda et al. [30] reported similar decreases in hardness in fish protein-enriched pasta, noting that this change may improve consumer acceptance. Likewise, researchers [16,17,20,21,33,34] observed reduced hardness and chewiness in pasta fortified with fish by-products.

Stickiness and cohesion showed distinct trends. Stickiness decreased with RT addition, which is likely related to the limited release of starch during cooking. Cohesiveness, however, remained relatively stable, indicating that fish protein contributed to maintaining structural integrity. Khodaei et al. [28] also found that fish protein fortification reduced stickiness while preserving cohesiveness, highlighting its contribution to the structural integrity of gluten-free pasta. In addition to starch dilution and protein matrix softening, possible interactions among trout protein, buckwheat starch, and xanthan gum may also contribute to these textural changes. Protein–starch complexes can alter gelatinization behavior, while protein–xanthan gum interactions (e.g., hydrogen bonding and electrostatic forces) may modify water distribution and gel structure, thereby influencing firmness and chewiness [1,6]. Springiness and elasticity behaved differently. Springiness, defined as the ability of the sample to recover its height after the first compression (calculated as the distance ratio between the two compressions), increased with RT supplementation. In contrast, elasticity, which reflects the structural integrity of the sample after deformation, slightly decreased compared to the control. The increase in springiness may be attributed to the ability of fish protein to form a network resembling gluten, while the reduction in elasticity highlights the limitations of this substitution. Calanche et al. [34] similarly reported that pasta enriched with fish by-products exhibited higher springiness but lower elasticity compared to traditional pasta. Gumminess was highest in the control sample and decreased with fish protein addition. This reduction suggests that fish protein interacts with starch to create a less resistant structure. Govinda et al. [30] confirmed that gumminess values decline in fish protein-supplemented pasta, aligning with the present findings.

### 3.3. Sensory Evaluation

The most significant barrier preventing consumers from accepting fish-enriched pasta is the “fishy odor.” Alcohols, aldehydes, ketones, esters, sulfur-containing compounds, and nitrogen-containing compounds (primarily amines) constitute the compounds responsible for the fishy odor in seafood [35,36,37]. In the present study, the fishy odor was eliminated by using vinegar, resulting in sensory scores close to the control group even at high fish content levels. In our study, the fishy odor was eliminated by using vinegar, resulting in sensory scores close to the control group even at high fish content levels. Acetic acid soaking primarily neutralizes volatile amines such as trimethylamine and dimethylamine by forming non-volatile amine salts, thereby reducing their release into the headspace [36,37]. In addition, the acidic environment can influence other odor-active compounds, including aldehydes, ketones, esters, and sulfur-containing volatiles, by altering their volatility or promoting acid-catalyzed reactions [38]. These combined effects explain the significant reduction in fishy odor intensity observed in sensory evaluation [39].

**Figure 2:**
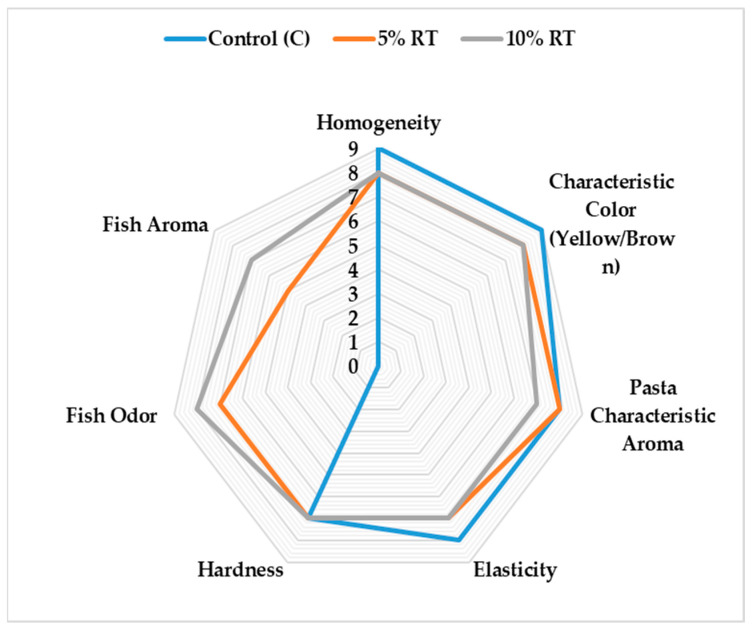
Sensory evaluation of gluten-free pasta enriched with odorless lyophilized rainbow trout powder (1 = dislike extremely, 9 = like extremely).

According to our analysis results, in terms of homogeneity and color, the control sample received the highest scores (9), while these values decreased slightly in samples with 5% and 10% trout content (Table 4, Figure 2). This can be explained by the fish powder creating particle heterogeneity in the dough structure and the natural pigments interacting with the buckwheat phenolic compounds, leading to darkening. However, the fact that the color scores are still at a “very good” level shows that the vinegar treatment preserves the visual stability of the fish powder. With respect to elasticity, the control sample exhibited higher values (8.00), whereas elasticity decreased slightly in the RT-enriched formulations (7.00). In contrast, hardness values remained constant across all samples (7.00), indicating that the incorporation of trout powder did not exert a measurable influence on this parameter. This can be explained by the fish protein, which replaces gluten, failing to form a sufficient elastic network in the dough structure. When evaluated based on the most important criterion, odor, the high fish odor scores (5%: 7; 10%: 8) prove the effectiveness of the vinegar treatment. The fish aroma was found to be moderate at a 5% addition (5.00) and more acceptable at a 10% addition (7). This indicates that the fish addition created a noticeable effect on the aroma, but it was considered acceptable by the panelists.

In studies on fish-added pasta, Monteiro et al. [22] reported that in tilapia flour-added pasta, fishy odor and canned fish taste scores dramatically decreased at rates above 12%. Pangestu et al. [3] reported that aroma and taste scores were significantly lower in sago pasta with tuna meal compared to the control group. Damat et al. [2] noted that gluten-free noodles containing fish meal were the least preferred formulations due to their strong odor and grayish color. Khodaei et al. [28] reported that although fish powder addition increased nutritional value, it was sensory unacceptable. These studies clearly demonstrate the sensory barrier of fish additives. However, the vinegar (acetic acid) treatment used in our study stands out as a unique method that overcomes this barrier. Vinegar protonates volatile amine compounds such as trimethylamine and dimethylamine, converting them into non-volatile salts, thereby preventing these compounds from escaping into the air and reducing odor perception. This mechanism not only reduces odor perception but also distinguishes itself from other methods in the literature by chemically binding and eliminating odor-causing components [4,37,38,39]. As a result, vinegar-based deodorization ensured sensory acceptability even at 10% trout inclusion, whereas other fish powders such as salmon or tilapia have frequently been reported as sensory unacceptable due to strong odor and flavor issues.

## 4. Conclusions

This study demonstrated that the combination of vinegar-based deodorization and lyophilization enables the successful incorporation of fish proteins into gluten-free pasta formulations. The enriched products showed significantly higher protein and mineral content compared to the control, while maintaining visual stability and improving protein digestibility.

The most distinctive contribution of this work lies in the sensory evaluation. Vinegar pretreatment effectively entrapped volatile compounds through acid–base neutralization, eliminating the characteristic “fishy odor” that typically limits consumer acceptance. Panelists confirmed that even at 10% fish inclusion, odor scores remained close to the control. Moreover, mean hedonic scores for taste and texture did not significantly differ between the control and RT-enriched samples, indicating that consumer perception of these attributes was maintained. This finding reversed the commonly reported trend in the literature of declining acceptance with increasing fish content. Panelists emphasized that the pasta was not only free from undesirable odor but also retained a pleasant flavor. Texture evaluation further showed that mouthfeel scores remained comparable to the control, confirming that the enriched samples were perceived as equally acceptable in terms of eating quality. This outcome underscores the success of the formulation strategy.

In conclusion, the integration of deodorized trout powder into gluten-free pasta provides a novel pathway to enhance nutritional quality without compromising sensory appeal. The positive response from panelists highlights the potential of this approach for industrial application and consumer acceptance, offering a promising strategy for the future development of gluten-free products.

## Figures and Tables

**Table 1:** Nutritional composition of gluten-free cooked pasta enriched with odorless lyophilized rainbow trout powder.

Applications	Moisture (%)	Protein %	Fat %	Ash %	Total Carbohydrates %	Energy Value kcal/100 g
Control (C)	34.05 ± 0.13 ^a^	11.82 ± 0.52 ^c^	0.65 ± 0.09 ^c^	0.76 ± 0.05 ^c^	52.71 ± 0.32 ^a^	264.4 ± 0.57 ^c^
5% RT	33.02 ± 0.20 ^b^	17.22 ± 0.36 ^b^	1.63 ± 0.04 ^b^	0.91 ± 0.03 ^ab^	47.21 ± 0.21 ^b^	272.42 ± 0.49 ^b^
10% RT	32.08 ± 0.11 ^b^	20.98 ± 0.09 ^a^	2.46 ± 0.05 ^a^	0.93 ± 0.05 ^a^	43.54 ± 0.24 ^c^	280.25 ± 0.78 ^a^

^a, b, c^: Values within the same column followed by different letters are significantly different (*p* < 0.05).

**Table 2:** Impact of odor-free lyophilized rainbow trout powder on technological and nutritional properties of gluten-free pasta.

	Weight Increase (%)	Volume Increase (%)	Swelling Index (g/g)	Cooking Loss (%)	Cooking Time (min, sn)	Glycemic Response (%)	In Vitro Protein Digestibility (%)
Control (C)	114.84 ± 6.79 ^a^	155.56 ± 1.14 ^a^	1.94 ± 0.04 ^a^	4.06 ± 0.18	6 min 13 s ± 0.04 ^c^	61.21 ± 0.81 ^a^	76.79 ± 1.02 ^c^
5% RT	108.68 ± 1.34 ^b^	149.4 ± 8.19 ^b^	1.93 ± 0.08 ^a^	4.10 ± 0.05	6 min 45 s ± 0.05 ^b^	53.30 ± 0.65 ^b^	79.57 ± 3.56 ^b^
10% RT	100.51 ± 2.1 ^c^	145.29 ± 2.37 ^b^	1.84 ± 0.10 ^b^	4.16 ± 0.12	7 min 48 s ± 0.09 ^a^	47.28 ± 2.35 ^c^	84.34 ± 3.51 ^a^

^a, b, c^: Values within the same column followed by different letters are significantly different (*p* < 0.05).

**Table 3:** Texture profile analysis (TPA) parameters of gluten-free pasta samples.

	Hardness (g)	Stickiness (g·s)	Springiness	Cohesion	Gumminess	Chewiness (mJ)	Elasticity
Control (C)	993.45 ± 10.0 ^a^	−0.93 ± 0.02 ^b^	14.62 ± 0.11 ^c^	0.76 ± 0.02 ^a^	751.45 ± 14.52 ^a^	10,965.10 ± 20.45 ^a^	1.28 ± 0.08 ^a^
5% RT	756.53 ± 8.52 ^b^	−0.59 ± 0.02 ^a^	20.89 ± 0.04 ^b^	0.76 ± 0.04 ^a^	533.34 ± 8.12 ^b^	10,204.72 ± 20.04 ^b^	1.21 ± 0.05 ^b^
10% RT	705.48 ± 10.0 ^c^	−0.57 ± 0.01 ^a^	21.01 ± 0.08 ^a^	0.71 ± 0.09 ^b^	510.31 ± 11.30 ^c^	9554.12 ± 18.36 ^c^	1.06 ± 0.07 ^c^

^a, b, c^: Values within the same column followed by different letters are significantly different (*p* < 0.05).

**Table 4:** Median sensory scores of pasta samples with different RT levels.

	Homogeneity	Characteristic Color (Yellow/Brown)	Pasta Characteristic Aroma	Elasticity	Hardness	Fish Odor	Fish Aroma
Control (C)	9 ^a^	9 ^a^	8 ^a^	8 ^a^	7 ^a^	0	0
5% RT	8 ^b^	8 ^a^	8 ^a^	7 ^b^	7 ^a^	7 ^b^	5 ^b^
10% RT	8 ^b^	8 ^a^	7 ^b^	7 ^b^	7 ^a^	8 ^a^	7 ^a^

^a, b^: Values within the same column followed by different letters are significantly different (*p* < 0.05).

## Data Availability

The original contributions presented in the study are included in the article, further inquiries can be directed to the corresponding author.
